# XIAP Deficiency Impairs Colonic Tuft Cell Development and Predisposes to Crohn's Disease

**DOI:** 10.1002/mco2.70745

**Published:** 2026-04-25

**Authors:** Rongli Fang, Wei Wang, Li Zhang, Jiwei Huang, Lei Huang, Huibo Wu, Yuyin Qi, Lili Li, Liren Tan, Min Zhang, Jianheng Zhu, Xiang Peng, Kanghua Zhong, Ming Zou, Xi Yang, Qiuhua Wang, Changjun Nie, Chaorui Tang, Ning Tang, Lanlan Geng, Hanhan Chen, James E. Vince, Hirokazu Kanegane, Xiaodong Zhao, Huifang Xian, Wenhao Zhou, Min Zhi, Yuxia Zhang, Zhanghua Chen

**Affiliations:** ^1^ Clinical Research Center For Pediatric Infection and Immunity and Department of Gastroenterology Guangzhou Women and Children's Medical Center Guangzhou Medical University Guangzhou China; ^2^ Department of Comprehensive Experimental Center Guangxi Clinical Research Center for Obstetrics and Gynecology Liuzhou Hospital of Guangzhou Women and Children's Medical Center Liuzhou China; ^3^ Department of Gastroenterology and Biomedical Innovation Center The Sixth Affiliated Hospital Sun Yat‐Sen University Guangzhou China; ^4^ Department of Rheumatology and Immunology Children's Hospital of Chongqing Medical University Chongqing China; ^5^ The Walter and Eliza Hall Institute of Medical Research and the Department of Medical Biology University of Melbourne Parkville, Vic Australia; ^6^ Department of Child Health and Development Graduate School of Medical and Dental Sciences Tokyo Medical and Dental University (TMDU) Tokyo Japan

**Keywords:** Crohn's disease, JAK inhibition, tuft cell, Wnt/β‑catenin–ASCL2, XIAP deficiency

## Abstract

A substantial proportion of patients with X‑‐linked inhibitor of apoptosis (XIAP) deficiency develop severe and treatment‑‐refractory Crohn's disease (CD). Although hematopoietic stem cell transplantation (HSCT) remains the only curative option for these patients, its outcomes are suboptimal, with a long‑‐term survival rate of only 50%. Therefore, identifying novel therapeutic targets is crucial to bridge this unmet clinical need. Here, we demonstrate that the abundance of tuft cells is reduced in both XIAP‐deficient CD patients and *Xiap* knockout (*Xiap* ^−/−^) mice. Mechanistically, XIAP deficiency reduces TLE4 ubiquitination, resulting in elevated TLE4 protein levels and consequent suppression of Wnt/β‑‐catenin–ASCL2 signaling, which is critical for secretory lineage differentiation. Tuft cell deficiency may increase susceptibility to microbial dysregulation, thereby promoting intestinal inflammation. Furthermore, we demonstrate that JAK inhibition promotes tuft cell regeneration and ameliorates mucosal inflammation in *Xiap* ^−/−^ mice. Consistently, in an XIAP‑‐deficient CD patient, treatment with a selective JAK1 inhibitor effectively increased tuft cell proportion and alleviated colonic symptoms. In conclusion, our study identifies tuft cell deficiency as a trigger of intestinal pathology in XIAP‑‐deficient Crohn's disease and suggests JAK inhibition as a promising therapeutic strategy.

## Graphical abstract

1. XIAP deficiency leads to impaired colonic tuft cells development and JAK–‐STAT pathway hyperactivation in CD patients and mice.
2. Mechanically, XIAP regulates tuft cells development through Wnt–‐TLE4/TCF–‐ASCL2 signaling.
3. Tuft cells deficiency predisposes XIAP‑‐deficient CD patients or *Xiap* ^−/−^‐/‐ mice to hazardous microbial effects, driving colonic inflammation.
4. JAK inhibition to promote tuft cells generation ameliorates colonic mucosal inflammation in *Xiap* ^−/−^‐/‐ mice and XIAP‑‐deficient CD patient.
5. Above findings shed light on the pathogenesis of tuft cells disordered development and reveal the potentially targets for the diagnosis and treatments for XIAP‑‐deficient CD patients.

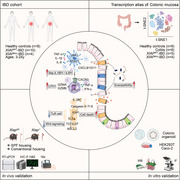

## 1 Introduction

The intestinal epithelium consists of stem cells, transient amplifying progenitor cells, absorptive enterocytes (ECs), and secretory lineages (including goblet, enteroendocrine, Paneth, and tuft cells). [1] Secretory cells contribute to the formation of chemical barriers, primarily through the production of mucin glycoproteins by goblet cells [2, 3] and antimicrobial peptides secreted by Paneth cells. [4] Tuft cells are chemosensory epithelial cells that line the intestinal epithelium, where they detect luminal substances and relay signals to neuronal cells. [5] They also play a critical role in antihelminth immunity by secreting IL‑‐25 and leukotrienes, which activate Type 2 immune responses. [6, 7] Together, the chemical and physical barriers established by epithelial cells collectively prevent microbial translocation, segregate commensal microbes from underlying immune cells, and maintain intestinal homeostasis. Disruption of the mucosal barrier is the key pathogenic mechanism in inflammatory bowel disease (IBD), including ulcerative colitis, and Crohn's diseases (CD). [8]

Wnt signaling is essential for intestinal stem cell self‑‐renewal and differentiation into secretory lineages. [9] Wnt pathway activation leads to β‑‐catenin stabilization and its association with T‑‐cell factor/lymphoid enhancer factor (TCF/LEF) to activate transcription, whereas in the absence of Wnt signaling, Groucho (Gro)/TLE corepressors bind TCF/LEF to suppress Wnt‑‐responsive genes. [10] The stability and activity of Gro/TLE are regulated posttranslationally via ubiquitination. X‑‐linked inhibitor of apoptosis (XIAP), a well‑‐characterized E3 ubiquitin ligase, mediates the ubiquitination and degradation of Gro/TLE, thereby promoting Wnt signaling. [11, 12] However, the role of XIAP in regulating intestinal stem cell dynamics and differentiation remains unclear.

XIAP deficiency (X‑‐linked lymphoproliferative syndrome Type 2, XLP2) [13], commonly causes severe colitis [14], and about 26% of the patients represented with refractory CD [15], for which hematopoietic stem cell transplantation (HSCT) is the only curative treatment. [16] The associated inflammation is complex and context dependent. NOD2 senses bacterial muramyl dipeptide (MDP) to activate NF‑‐κB and MAPK signaling, a process requiring XIAP‑‐mediated ubiquitination of RIPK2. [17] Consequently, XIAP deficiency impairs MDP‑‐induced inflammatory responses in myeloid cells. [18] Paradoxically, XIAP‑‐deficient macrophages show enhanced NLRP3 inflammasome activation and increased IL‑‐1β/TNF‑‐α secretion. [19, 20, 21] Loss of XIAP in myeloid cells promotes caspase‑‐8 (CASP8)‑‐ and RIPK3‑‐dependent apoptosis and pyroptosis. [18, 20, 22, 23] In *Xiap* knockout (*Xiap^−/−^*) mice, reduced Paneth and dendritic cell numbers resulting from aberrant TLR5/TNFR1/2 signaling contribute to dysbiosis and spontaneous ileitis [24, 25].

Nevertheless, whether XIAP deficiency promotes inflammation by affecting intestinal epithelial barrier function remains unclear, and the mechanisms underlying intestinal inflammation and enterocyte dysfunction in XIAP‑‐deficient CD are still poorly understood. Here, by profiling the single‑‐cell landscape of colonic mucosa from four XIAP‑‐deficient CD patients, along with in vitro and in vivo validation experiments and a therapeutic trial in one patient, we identified a therapeutic candidate that ameliorated epithelial defects caused by XIAP deficiency. These findings not only provide mechanistic insights into how XIAP affects intestinal tuft cells to drive inflammation, but also suggest a potential new therapeutic strategy for XIAP‑‐deficient CD patients.

## 2 Results

### 2.1 Identification of XIAP Variants in Pediatric and Refractory IBD Patients

**FIGURE 1:**
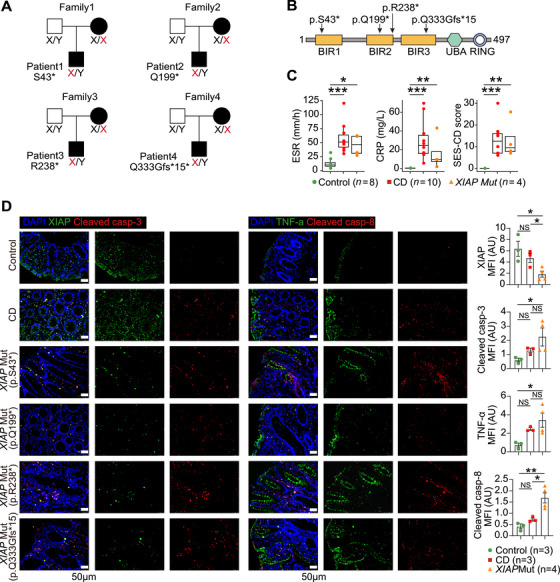
Identification and functional evaluation of *XIAP* variants. (A) Identified *XIAP* variants in four patients, with mutant alleles shown in red. (B) Schematic representation of four truncating variants across XIAP functional domains. (C) Box plots comparing ESR, CRP, and SES‑‐CD score among control subjects, non‑‐XIAP‑‐deficient, and XIAP‑‐deficient CD patients. Boxes indicate the 25% quantile, median, and 75% quantile. *Abbreviations*: ESR, erythrocyte sedimentation rate; CRP, C‑‐reactive protein; SES‑‐CD, simplified endoscopic score for CD. (D) Representative immunofluorescence images and bar plots analysis of XIAP, cleaved caspase‑‐3, TNF‑‐α, and cleaved caspase‑‐8 in colonic tissues across groups. *Abbreviations*: MFI, mean fluorescence intensity. Data were expressed as mean ± SEM. *p* Values were calculated using the Mann–Whitney *U*‑‐test (C) and one‑‐way ANOVA (D). *p < 0.05, **p < 0.01, ***p < 0.001, NS not significant.

We performed whole‑‐exome sequencing (WES) in pediatric refractory IBD patients and their unaffected parents, identifying four male CD patients with *XIAP* variants (Figure 1A). Patients 1–3 carried nonsense variants (p. S43*, p.Q199*, and p. R238*), and Patient 4 had a frameshift variant (p. Q333Gfs*15) (Figure 1B). Inflammatory markers C‑‐reactive protein (CRP) and erythrocyte sedimentation rate (ESR) and simplified endoscopic score for CD (SES‑‐CD) were significantly elevated in CD patients versus controls, but did not differ between patients with and without *XIAP* variants (Figure 1C). Immunofluorescence staining showed decreased XIAP expression and increased CASP8 activation in colonic biopsies from these four patients with *XIAP* variants (Figure 1D). These results confirm that the identified variants cause XIAP loss and impair its anticell death function in the colonic mucosa of affected patients.

### 2.2 The Abundance of Tuft Cells is Reduced in the Colonic Mucosa of Both XIAP‑‐Deficient CD Patients and *Xiap^−/−^* Mice

**FIGURE 2:**
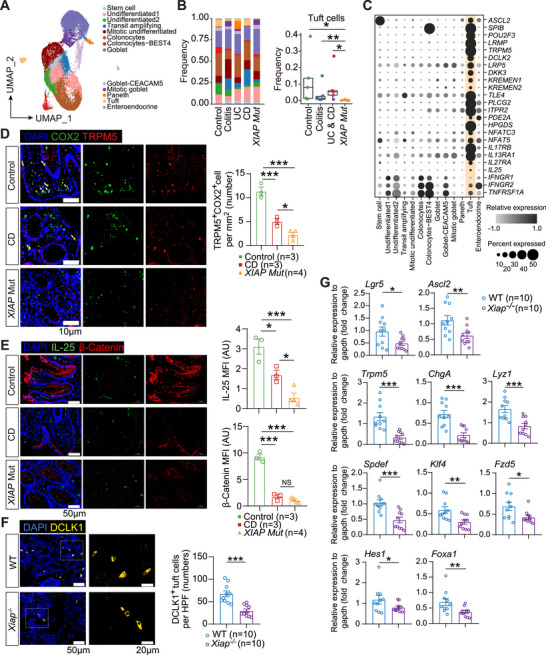
XIAP deficiency is associated with reduced tuft cell abundance. (A) UMAP visualization of 13 epithelial cell subsets. (B) Left, bar plots showing epithelial subset compositions across groups. Right, box plots comparing the frequency of tuft cells in control (*n* = 6), colitis (*n* = 6), IBD (*n* = 5), and XIAP‐deficient CD (*n* = 4) groups. Boxes indicate the 25% quantile, median, and 75% quantile. (C) Dot plots displaying tuft cell markers expression across epithelial subsets. (D and E) Immunofluorescence staining comparing the number of TRPM5^+^COX2^+^ tuft cells (D), β‑‐catenin and IL‑‐25 (E) expressions in colonic mucosal tissues across groups. (F and G) Immunofluorescence staining assessing DCLK1^+^ tuft cell numbers (F), and bar plots comparing mRNA expression levels of indicated genes (G), in the colonic mucosa of WT and *Xiap^−/−^* mice under SPF housing conditions. Data were expressed as mean ± SEM. *p* Values were calculated using the Mann–Whitney *U*‑‐test (B), one‑‐way ANOVA (D and E) and Student's *t*‑‐test (F and G). *p < 0.05, **p < 0.01, ***p < 0.001.

We performed single‑‐cell RNA sequencing (scRNA‑seq) on colonic mucosal tissues from four XIAP‑deficient CD patients and compared the data with those from six healthy controls, six chronic colitis patients, and five IBD patients with normal XIAP expression. [26] The detailed characteristics of the cohort are summarized in Table S1. Notably, analysis of colonic epithelial cells revealed a marked reduction in tuft cell numbers within the mucosa of XIAP‑deficient CD patients compared with healthy controls and other patient groups (Figure 2A,B). Tuft cells specifically express transcription factor genes (*ASCL2*, *SPIB*, *POU2F3*), Wnt signaling pathway genes (*LRP5*, *DKK3*, *KREMEN1*/*2*, *TLE4*), and Th2 immune response‐associated genes (*IL17RB*, *IL13RA1*, *IL25*) (Figures 2C and S1). [27, 28, 29] Immunofluorescence staining further confirmed a significant reduction in TRPM5^+^COX2^+^ tuft cells [30, 31], along with decreased levels of secreted IL‑‐25 and β‑‐catenin, in XIAP‑‐deficient CD patients compared with healthy controls and other CD patients (Figure 2D,E).

To validate XIAP's role in tuft cell development, we generated *Xiap^−/−^* and wild‑type (WT) littermates and housed them under specific pathogen‑‐free (SPF) conditions. Immunofluorescence showed significantly fewer DCLK1^+^ tuft cells in the colonic mucosa of *Xiap^−/−^* mice (Figure 2F). Key genes related to colonic stemness (*Lgr5*, *Spdef*, *Klf4*), Wnt signaling (*Ascl2*), and secretory lineage markers (*Trpm5*, *Chga*, *Lyz1*) were consistently downregulated in *Xiap* ^−/−^ mice (Figure 2G). Organoids derived from *Xiap^−/−^* colonic crypts and cultured under secretory differentiation conditions also showed reduced expressions of both *Xiap* and tuftcell markers (*Pou2f3*, *Ascl2*, *Dclk1*, *Trpm5*), as well as fewer DCLK1^+^ tuft cells, compared with organoids from WT mice (Figure S2A,B). These results collectively indicate that XIAP deficiency impairs tuftcell differentiation.

### 2.3 XIAP Regulates Tuft Cell Development via the Wnt/β‑‐Catenin–ASCL2 Signaling Pathway

XIAP has been reported to activate Wnt signaling by inducing TLE degradation, which relieves TCF/LEF‑mediated transcriptional repression. [12] Activated Wnt signaling upregulates ASCL2, a key regulator of intestinal stem and secretory lineages. [32, 33] We therefore hypothesized that XIAP directly regulates tuft‑cell differentiation.

**FIGURE 3:**
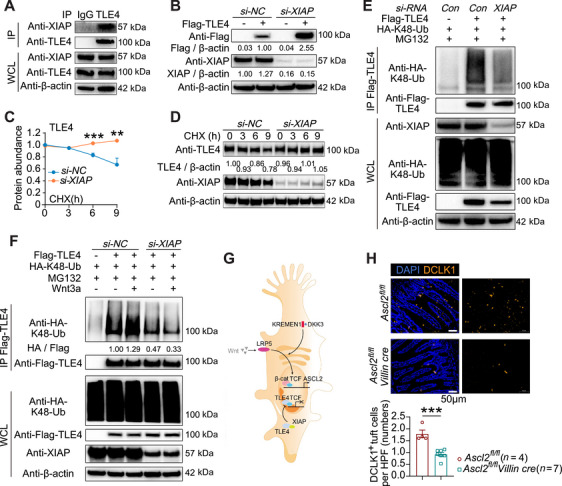
XIAP regulates tuft cell development via the Wnt–TLE4/TCF–ASCL2 axis. (A) Co‑IP and western blotting showing direct XIAP–TLE4 interaction in HEK293T cells. (B) Western blotting showing increased TLE4 expression in *si‑‐XIAP*‑‐transfected Caco‑‐2 cells relative to *si‑‐NC* controls. (C and D) Line charts (C) and western blotting (D) displaying time‑dependent TLE4 levels in *si‑‐NC* and *si‑‐XIAP*‑‐transfected HEK293T cells after CHX treatment (100 µg/mL) for 3, 6, and 9 h. Experiments were performed with two biological replicates. (E) Co‑‐IP and western blotting revealing decreased binding of Flag–TLE4 to Ub‑K48 chain in *si‑‐XIAP*‑‐transfected HEK293T cells compared with *si‑‐NC* controls. (F) Wnt3a (200 ng/mL, 24 h) enhancing Flag–TLE4 and HA–K48–Ub association in HEK293T cells. (G) Schematic representation of the tuft‑cell‑related genes in Wnt/β‑catenin–ASCL2 signaling. (H) Immunofluorescence staining comparing DCLK1^+^ tuft cell numbers in the colonic mucosa of *Ascl2* ^fl/fl^ villin‑‐cre and littermate controls. Data were expressed as mean ± SEM. *p* Values were calculated using two‑‐way ANOVA (C) and Student's *t*‑‐test (H). **p < 0.01, ***p < 0.001.

To test this, we first assessed XIAP's regulatory effect on TLE4, a TLE family member highly expressed in tuft cells. Co‑immunoprecipitation (Co‑‐IP) confirmed a direct XIAP–TLE4 interaction (Figure 3A). *XIAP* knockdown increased TLE4 protein levels under basal conditions and delayed its degradation upon cycloheximide (CHX) treatment (Figure 3B–D). Since K48‑linked polyubiquitination typically directs proteasomal degradation [12, 34], we confirmed that *XIAP* knockdown reduced K48‑linked ubiquitination of TLE4 (Figure 3E). Given that XIAP binds and ubiquitinates TLE4, we then investigated whether Wnt signaling enhances this process. Treatment with the Wnt signaling agonist (Wnt3a) increased TLE4 ubiquitination (Figure 3F). Together, these findings show that XIAP restricts TLE4 protein levels via ubiquitin‑mediated proteasomal degradation (Figure 3G).

To confirm whether *TLE4* knockdown could rescue tuft cell development, we infected *Xiap^−/−^* colonic organoids with *Tle4*‑targeting adenovirus. *Tle4* knockdown increased *Ascl2* and *Dclk1* (marker gene of tuft cells) expression compared with untreated *Xiap^−/−^* organoids (Figure S2C,D). Similarly, the TLE4 inhibitor DAPT downregulated *Tle4* and upregulated *Dclk1* and *Ascl2* in *Xiap^−/−^* organoids (Figure S2E). These results demonstrate that reducing TLE4 activity compensates for XIAP loss in secretory epithelial cells. Epithelial‑specific *Ascl2* knockout mice (*Ascl2* ^fl/fl^ villin‐cre) housed under SPF conditions also showed a marked reduction in DCLK1^+^ tuft cells compared with *Ascl2* ^fl/fl^ controls (Figure 3H), further underscoring the essential role of the Wnt–‑ASCL2 axis in tuft cell development.

### 2.4 Reduced Tuft Cells Due to XIAP Deficiency Predispose to Colonic Inflammatory Responses

**FIGURE 4:**
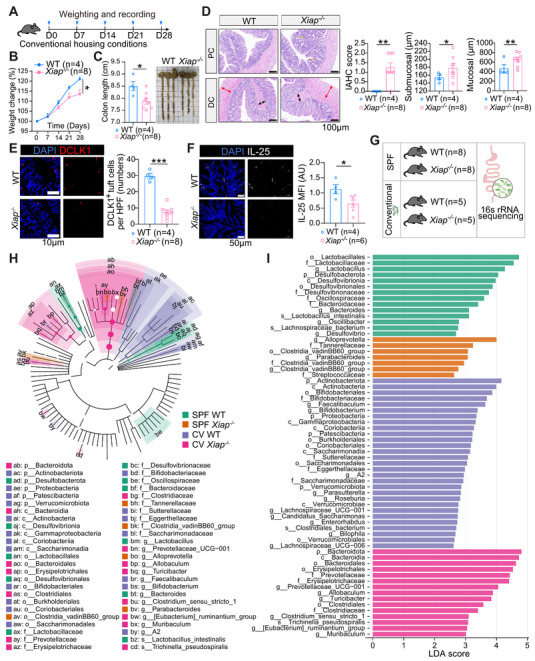
*Xiap^−/−^* mice exhibit spontaneous colitis under conventional housing conditions. (A) Experimental timeline for conventionally housed WT and *Xiap^−/−^* mice. (B and C) Body weight changes (B) and colon length comparison (C) between WT and *Xiap^−/−^* mice. (D) Representative H&E‑‑‐stained colon sections from WT and *Xiap^−/−^* mice. Bar plots comparing IAHC scores, submucosal thickness, and muscular layer thickness. *Abbreviations*: PC, proximal colon. DC, distal colon. (E and F) Immunofluorescence analysis of DCLK1^+^ tuft cell abundance (E), and expression levels of IL‑‐25 (secreted) (F) in the colonic mucosa of WT and *Xiap^−/−^* mice under conventional housing conditions. (G) Group allocation and mouse numbers for 16S rRNA sequencing. (H) Cladogram of taxonomic differences from phylum to genus/species. Node size reflects relative abundance. (I) Linear discriminant analysis (LDA) scores (>2.5) of differentially abundant taxa across groups. Data were presented as mean ± SEM. Statistical analyses were performed using the two‑‐way ANOVA (B), Student's *t*‑‐test (C–F). *p < 0.05, **p < 0.01, ***p < 0.001.

To determine how XIAP‑dependent tuft‑cell loss promotes colonic inflammation, we first compared *Xiap^−/−^* and WT mice under SPF conditions. No significant differences in body weight, colon length, IAHC (inflammation‑‐associated histological changes) scores, or edema were observed, indicating no overt colitis (Figure S3A–D). However, under conventional housing, *Xiap^−/−^* mice spontaneously developed colitis, displaying reduced body weight, shortened colon length, elevated IAHC scores, and increased submucosal/muscular thickening (Figure 4A–D). Immunofluorescence confirmed marked reductions in both DCLK1^+^ tuft cells and secreted IL‑25 in *Xiap^−/−^* colonic mucosa (Figure 4E,F).

We then compared colonic microbiota in WT and *Xiap^−/−^* mice under SPF or conventional housing conditions (Figure 4G). XIAP deficiency reduced α‑diversity under both conditions and markedly altered β‑diversity (Figure S3E,F), indicating consistent microbial dysbiosis. Liner discriminant analysis effect size analysis showed distinct taxa changes in *Xiap^−/−^* mice. Under conventional housing, these mice showed decreased beneficial Bifidobacterium and Firmicutes and increased Bacteroidota (Figure 4H,I). Reduced Firmicutes levels have previously been linked to the pathogenesis of CD. [35, 36] At the genus level, *Xiap^−/−^* mice exhibited elevated Allobaculum and Prevotellaceae, two taxa associated with epithelial adherence, mucus degradation, and intestinal inflammation. [37, 38] Taken together, *Xiap^−/−^* mice developed spontaneous colitis under conventional, not SPF housing, indicating that XIAP deficiency alone is insufficient to induce inflammation. Instead, the associated loss of tuft cells and dysbiosis likely increase colonic mucosal susceptibility to pathogens, triggering inflammation upon environmental exposure.

### 2.5 The Colonic Mucosa of XIAP‑‐Deficient CD Patients Exhibits Activated JAK–STAT Signaling

**FIGURE 5:**
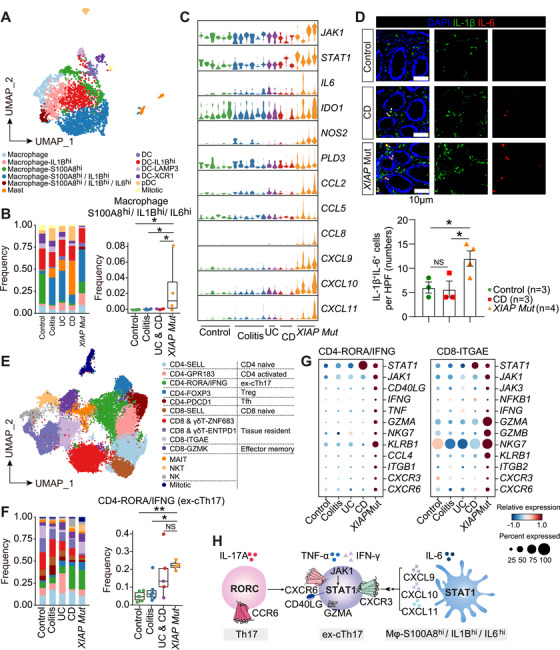
Immune profiling indicates activated JAK–STAT signaling in XIAP‑deficient CD. (A) UMAP plot of 12 myeloid subsets. (B) Left, bar plots showing myeloid subset compositions across groups. Right, box plots comparing the frequency of IL‑‐1β^+^IL‑‐6^+^ inflammatory macrophages in control (*n* = 6), colitis (n = 6), IBD (*n* = 5), and XIAP‑‐deficient CD (*n* = 4) groups. Boxes indicate the 25% quantile, median, and 75% quantile. (C) Violin plots comparing expression levels of marker genes in *S100A8*/*A9* ^+^ *IL1B* ^+^ *IL6* ^+^ inflammatory macrophages across groups. (D) Immunofluorescence staining comparing the number of IL‑‐1β^+^IL‑‐6^+^ cells (white arrows indicated) in the colonic mucosa across groups. (E) UMAP plot depicting 16 T and NK cell subsets. (F) Left, bar plots showing T and NK subset compositions across groups. Right, box plots comparing the frequency of IFN‑‐γ^+^ ex‑‐Th17 T cells in control (*n* = 6), colitis (*n* = 6), IBD (*n* = 5), and XIAP‑‐deficient CD (*n* = 4) groups. (G) Dot plots showing Th1‑signature gene expressions in two IFN‑γ^+^ T subsets. (H) Schematic representation of the reciprocal *CXCL9*/*10*/*11* (macrophages) and *CXCR3*/*6* (T cells) expression suggests potential direct interaction. Data were expressed as mean ± SEM. *p* Values were calculated using the Mann–Whitney *U*‑‐test (B and F) and one‑‐way ANOVA (D). *p < 0.05, **p < 0.01, NS not significant.

We next profiled immune responses in colonic mucosa from XIAP‑deficient CD patients. Analysis of 12 myeloid subsets showed *S100A8*/*A9* ^+^ *IL1B* ^+^ inflammatory macrophages enriched in all disease groups (Figures 5A and S4A,B). Notably, a distinct subset (*S100A8*/*A9* ^+^ *IL1B* ^+^ *IL6* ^+^ inflammatory macrophages) was elevated specifically in XIAP‑deficient patients and expressed higher levels of *IL6*, *JAK1*, *STAT1*, *IDO1*, *NOS2*, and chemokines *CCL2*/*5*/*8* and *CXCL9*/*10*/*11* (Figure 5B,C). Immunofluorescence analysis further confirmed a significant increase in IL‑‐1β^+^IL‑‐6^+^ cells in these patients compared with controls, colitis patients, and other IBD patients (Figure 5D).

T‑cell subsets were also altered in XIAP‑deficient patients (Figures 5E and S4C,D). Among 16 T/NK/NKT subpopulations analyzed, two IFN‑‐γ^+^ T cell subsets (*RORC* ^+^ *IL17A* ^+^ *IFNG* ^+^ ex‑‐cTh17 cells and *ITGAE* ^+^ tissue‑‐resident CD8 T cells) showed elevated expression of JAK–STAT components (*JAK1*, *STAT1*), proinflammatory cytokines (*IFNG*, *TNF*), lymphocyte cytotoxicity‑‐associated genes (*GZMA*/*B*, *NKG7*), and chemokine receptors genes (*CXCR3*/*6*) (Figure 5F,G). Notably, chemokines *CXCL9*/*10*/*11* (expressed by IL‑‐1β^+^IL‑‐6^+^ macrophages) and their receptors *CXCR3*/*6* (expressed by ex‑cTh17 cells) were both upregulated, accompanied by JAK–STAT activation in these subsets (Figure 5H).

To validate JAK–STAT activation in XIAP‑deficient CD, we analyzed two intestinal mucosa bulk RNA‑seq datasets. [25, 39] The first showed downregulated tuft‑cell genes (*TRPM5*, *TRPM6*) and upregulated inflammatory macrophage markers (*IL6*, *IFNG*, *IL1B*, *CXCL8* and *CXCL10*) in XIAP‑deficient versus control and non‑‐XIAP deficient CD samples (Figure S5A,B). In the second dataset, molecular clustering identified a severe‑endoscopy‑enriched cluster with reduced expression of XIAP and tuft‑cell markers (*ASCL2*, *POU2F3*, *TRPM5*, *DCLK2*, *IL25*) and increased inflammatory/JAK–STAT genes (*IL6*, *IL1B*, *STAT1*, *CXCL9*, *CXCL10*) (Figure S5C–E). Deconvolution confirmed decreased tuft cells and increased *S100A8/A9* ^+^ *IL1B* ^+^ *IL6^+^* inflammatory macrophages in cluster4 (Figure S5F). Together, these results reinforce XIAP–‑tuft‑cell axis disruption to JAK–STAT activation.

### 2.6 JAK Inhibition Restores Colonic Tuft Cells in *Xiap* ^−/−^ Mice

**FIGURE 6:**
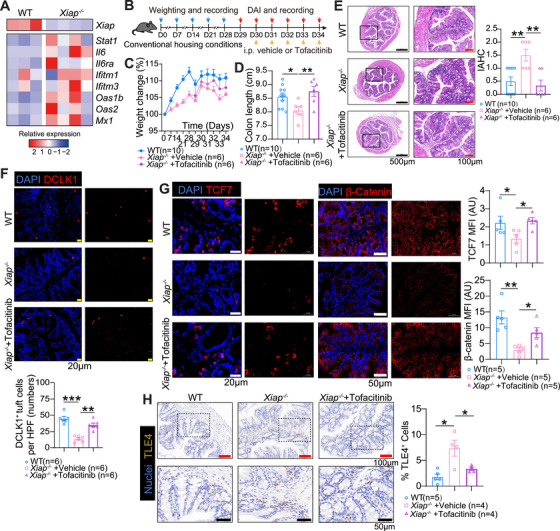
JAK inhibition restores colonic tuft cells in *Xiap^−/−^* mice. (A) Heatmap showing *Xiap* and interferon‑related gene expressions in colon tissues of conventionally housed WT and *Xiap^−/−^* mice. (B) Experimental timeline: 6‑week‑old WT and *Xiap^−/−^* mice were transferred from SPF to conventional housing. Arrows indicate start of tofacitinib (15 mg/kg/day, i.p.) treatment. (C and D) Body weight changes (C) and colon length comparison (D) between WT and *Xiap^−/−^* mice with or without tofacitinib treatment. (E) Representative H&E‑‐stained colon sections from WT and *Xiap^−/−^* mice with or without treatment. Bar plots comparing IAHC scores. (F and G) Immunofluorescence staining and bar plots comparing DCLK1^+^ tuft cell abundance (F), TCF7 and β‑‐catenin expressions (G), in the colonic mucosa from WT and *Xiap^−/−^* mice with or without treatment. (H) Immunohistochemistry (IHC) analysis comparing the percentage of TLE4^+^ cells in the colonic mucosa of WT and *Xiap^−/−^* mice with or without treatment. Data were expressed as mean ± SEM. Statistical analyses were performed using the two‑‐way ANOVA (C) and one‑‐way ANOVA (D–H). *p < 0.05, **p < 0.01, ***p < 0.001.

We next investigated JAK–STAT pathway activity in conventionally housed *Xiap^−/−^* mice, which showed elevated expression of interferon‐response genes (*Ifitm1*, *Ifitm3*, *Oas1b*, *Oas2*, *Mx1*), *Il6*, and *Il6ra*, confirming pathway activation compared with WT mice (Figure 6A). We then tested whether JAK–STAT inhibition could alleviate colitis (Figure 6B). *Xiap^−/−^* mice treated with the pan‐JAK inhibitor tofacitinib displayed increased body weight and colon length, improved histopathology, and reduced IAHC scores compared with untreated *Xiap^−/−^* mice (Figure 6C–E). Importantly, tofacitinib treatment significantly increased DCLK1^+^ tuft cell numbers and upregulated β‑‐catenin and TCF7 while downregulating TLE4 in colon biopsies (Figure 6F–H). These results indicate that JAK inhibition restores tuft cells by modulating the Wnt–TLE4/TCF signaling axis.

Next, we tested whether restoring tuft cells could ameliorate inflammation by administering succinate or recombinant mouse IL‑‐25 (rmIL‑‐25) to *Xiap^−/−^* mice. [28] Both treatments significantly increased DCLK1^+^ tuft cell numbers in colon biopsies, improved colon length, and reduced IAHC histopathology scores compared with untreated mice (Figure S6A–D). These results confirm the essential role of tuft cells in alleviating colitis in XIAP deficiency.

### 2.7 JAK Inhibition Alleviates Clinical Symptoms in an XIAP‑‐Deficient CD Patient

**FIGURE 7:**
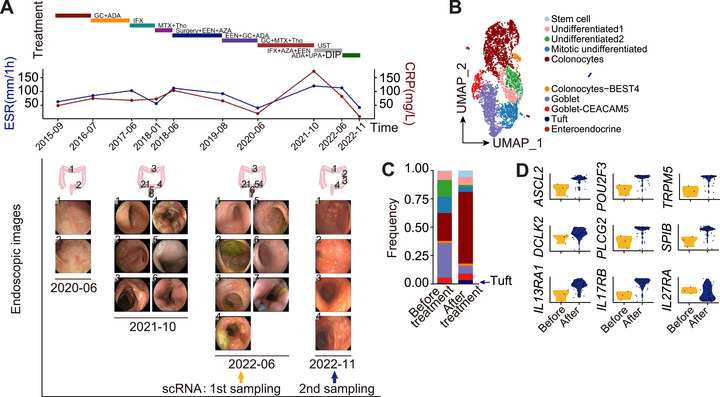
JAK inhibitor alleviates clinical symptoms in a XIAP‑‐deficient CD patient. (A) Schematic representation of the treatment progression (top panel), ESR and CRP measurement results (middle panel), and colonic endoscopic images (bottom panel) at different treatment points, and scRNA‑‐seq sampling points before/after upadacitinib treatment. *Abbreviations*: GC, glucocorticoid; ADA, adalimumab; IFX, infliximab; MTX, methotrexate; Tho, thalidomide; EEN, exclusive enteral nutrition; AZA, azathioprine; UST, ustekinumab; UPA, upadacitinib; DIP, dipyridamole. (B) UMAP plot of 10 epithelial cell subsets in the XIAP‑‐deficient CD patient before and after treatment. (C and D) Bar plots showing the cellular proportions of epithelial cell subsets (C), and violin plots comparing expression levels of tuft cell marker genes in the XIAP‑‐deficient CD patient before and after treatment.

We recruited Patient 1, a 29‑‐year‑‐old male with chronic CD due to XIAP‑‐deficient (p.S43*), for precision‐guided therapy. He had an 8‑‐year history of severe CD, with recurrent flares and complications despite multiple treatments, including corticosteroids, biologics (adalimumab, infliximab, vedolizumab, ustekinumab), immunosuppressants (azathioprine, methotrexate, thalidomide), and surgery. His disease featured intestinal ulcers, transmural inflammation, and perianal involvement, showing only transient, partial responses—highlighting a refractory, relapsing course. Informed by multiomics and preclinical data, a personalized regimen of adalimumab, upadacitinib (JAK1 inhibitor), and phosphodiesterase inhibitor dipyridamole was initiated. After 5 months, he achieved sustained clinical remission with reduced inflammatory markers (ESR, CRP) and improved mucosal healing (Figure 7A).

ScRNA‑‐seq of the patient's colonic mucosa after personalized therapy showed a higher tuft cell proportion and upregulated expressions of *ASCL2*, *POU2F3*, *TRPM5*, and *DCLK2* (Figure 7B–D). In contrast, inflammatory signatures, including genes linked to IL‑‐1β^+^IL‑‐6^+^ inflammatory macrophages and IFN‑‐γ^+^ ex‑‐cTh17 T cells (*STAT1*, *JAK1*, *IL6*, *TNF*, *IL1B*, *CXCL10*), were markedly reduced (Figure S7A–D). Posttreatment analysis also revealed decreased TLE4^+^ cells and increased TCF7 expression (Figure S7E,F). These results suggest JAK–STAT inhibition as a potentially beneficial therapeutic strategy for XIAP‑‐deficient CD.

## 3 Discussion

Here, we performed scRNA‑‐seq analysis of four XIAP‑‐deficient CD patients and revealed a significant deficiency in colonic tuft cells. We further demonstrated that XIAP promotes tuft cell development by facilitating TLE4 degradation and activating the Wnt–ASCL2 signaling axis. Tuft cell deficiency directly impairs the intestinal barrier and promotes harmful bacterial colonization. For example, tuft cell‑‐deficient mice show reduced goblet cell responses and impaired infection control of *N.brasiliensis*. [40] A key mechanism involves the succinate receptor GPR91 on tuft cells; in their absence, succinate accumulates, driving dysbiosis by favoring succinate‑‐producing bacteria like *E. coli*. [41] Thus, tuft cell loss may increase the susceptibility of XIAP‑‐deficient patients to pathogenic microbes. Resulting microbial dysbiosis can activate inflammatory signaling via pattern recognition receptors, disrupting intestinal CD4^+^ T cell homeostasis and contributing to the pathogenesis of IBD. [42] In XIAP‑deficient CD patients, we observed macrophage hyperinflammation and a dominant IFN‑‐γ^+^ ex‑‐cTh17 cell response, both driven by activated JAK1–STAT signaling. In murine models of norovirus infection, tuft cells have been demonstrated to suppress Th1 immune responses and promote intestinal immunotolerance. [43] Thus, the exacerbated Th1 response observed in XIAP‑‐deficient CD patients may plausibly be associated with tuft cell deficiency. Supporting this, succinate supplementation to boost tuft cells has been shown to reduce inflammation in a TNF^ΔARE/+^ mouse model of CD‑‐like ileitis. [28, 44] Consistently, in *Xiap^−/−^* mice, restoring tuft cells with either succinate or IL‑25 alleviated colitis. Together, these findings highlight tuft cells have essential function in maintaining intestinal homeostasis and mitigating colonic pathology in XIAP deficiency.

Beyond known tuft cell regulators like POU2F3 and STAT6 [45, 46, 47], our work identifies a key role for the Wnt pathway in tuft cell development, mediated by β‑‐catenin, TCF/LEF, and their target gene ASCL2. [32, 33, 48, 49] Epithelial‑‐specific loss of ASCL2 reduces intestinal tuft cell numbers. The transcriptional repressor TLE4 inhibits β‑catenin/TCF binding and suppresses ASCL2. [10] By scRNA‑‐seq, we show that TLE4 and TCF7 are expressed at high levels in tuft cells. XIAP deficiency led to accumulation of TLE4 and downregulation of TCF7 and ASCL2. Paneth cells are reported as Wnt ligand secreting cells in the intestinal crypt, and Wnt signaling is itself essential for Paneth cell maturation. [50, 51] Together with our data, this highlights the broad, pivotal function of Wnt signaling in orchestrating the development of secretory epithelial cells, including both Paneth and tuft cells.

One unexpected finding was that JAK inhibition in *Xiap* ^−/−^ mice and an XIAP‑‐deficient CD patient reduced TLE4 but increased TCF7 expression, thereby enhancing Wnt/β‑catenin signaling and tuft cell differentiation. Although the exact mechanism remains unclear, our results suggest a regulatory network connecting JAK–STAT signaling, Wnt/β‑catenin activity, XIAP, and Gro/TLE repressors, offering potential strategies to modulate tuft cell numbers. We also explored the translational potential of JAK inhibition. In *Xiap* ^−/−^ mice, it suppressed inflammation and restored tuft cell numbers. In a patient case, JAK inhibitor therapy was initiated. Additionally, because XIAP‑deficient CD exhibits elevated *S100A8*/*A9* ^+^ *IL1B* ^+^ *IL6* ^+^ inflammatory macrophages with hyperactivated JAK–STAT signaling and high *IL1B*, *TNF*, and *PDE4B* expression, we combined treatment with the pan‑phosphodiesterase (PDE) inhibitor dipyridamole to target PDE4B and curb cytokine release. [26] This combination therapy reduced systemic inflammation and promoted mucosal healing with no observed adverse effects, providing preliminary support for further clinical investigation in XIAP‑deficient CD.

Our study has several limitations. First, scRNA‑‐seq data from only four XIAP‑‐deficient CD patients limit statistical power and generalizability. However, key findings that tuft cell loss and JAK–STAT dysregulation were validated in mouse models and public datasets. This supportive evidence suggests that the observations in these four patients are representative of XIAP‑‐deficient CD more broadly. Second, JAK inhibitor efficacy was assessed in a single patient, highlighting the need for multicenter randomized controlled trials with larger cohorts to comprehensively assess its long‑‐term efficacy, optimal dosing, and safety profile in this population. Nevertheless, for physicians managing refractory XIAP‑‐deficient CD patients who have exhausted conventional therapies, our data may provide preliminary evidence to consider JAK inhibition as a potential treatment option. Third, we are unable to determine the relative contributions of XIAP‑‐mediated pathogen surveillance, cell death, inflammation, and epithelial differentiation in promoting intestinal pathology in XIAP‐deficient CD patients.

In summary, we characterize hyperinflammation, IFN‑‐γ^+^ ex‑‐cTh17‐skewed immunity and tuft cell deficiency in XIAP‑‐deficient CD. We also show that JAK inhibition is a promising therapeutic strategy for XIAP deficiency, capable of restoring immune balance and promoting mucosal repair.

## 4 Materials and Methods

The detailed description of materials, methods, and primers sequences in this study are available in the Supporting Information.

### 4.1 Mice Experiments

*Xiap* knockout (*Xiap^−/−^*) and conditional knockout *Ascl2^flox/flox^* villin‑‐cre mice were generated and purchased from Shanghai Model Organisms Center, Inc. (Shanghai, China). All mice were maintained on a C57BL/6 genetic background and housed under SPF conditions until they reached 6–8 weeks of age.

Male *Xiap^−/−^* mice and WT littermates aged 6–8 weeks were transferred to conventional housing conditions for 4 weeks, with controlled ambient temperature (22°C) and humidity (55%). For experimental treatments, mice were administered separately with tofacitinib (MCE, i.p., 15 mg/kg/day, dissolved in 5% DMSO), succinate (MCE, daily in drinking water, 0.02%), and recombinant IL‑‐25 (MCE, i.p., 300 µg/kg/day, dissolved in PBS) under conventional housing conditions. During the experimental period, body weight and general health status were monitored weekly to assess the development of intestinal symptoms. At the conclusion of the study, all experimental animals were humanely euthanized and organs were collected for subsequent analysis.

*Ascl2^flox/flox^* villin‑‐cre mice and littermate controls (6–8 weeks) were euthanized. Intestinal tissues were harvested and fixed for histopathological evaluation and immunofluorescence staining.

### 4.2 The Medical Condition of Patient 1 With XIAP Deficiency

The patient is a 29‑‐year‑‐old male with an 8‐year CD history presents with recurrent abdominal pain, diarrhea, and perianal discomfort, including fistulae and abscesses. Symptoms of this patient began in 2015, including abdominal pain, diarrhea, fever, perianal pain, oral ulcers, and knee arthralgia. Colonoscopy showed segmental inflammation and ulcers in the ileum, colon, and rectum. Initial treatment with corticosteroids and adalimumab resulted in symptomatic improvement, but recurred in July 2016. A repeat colonoscopy confirmed ulcer recurrence, prompting a switch to infliximab and azathioprine, to which the patient responded suboptimally. In June 2017, active disease was confirmed. The patient then received 2 months of exclusive enteral nutrition followed by 6 months of methotrexate and thalidomide, after which obstructive symptoms developed. Imaging identified stenosis at the ileocecal valve. In January 2018, the patient underwent surgery (partial resection of the terminal ileum, right hemicolectomy, and ileocolic anastomosis), with histopathology confirming CD. Postoperatively, total enteral nutrition and azathioprine were initiated, but azathioprine was later discontinued due to respiratory and urinary tract infections. In June 2018, prednisone and adalimumab induced remission, but symptoms recurred after 1 year. Imaging confirmed recurrent ulcers and new perianal fistulae/abscesses, indicating disease reactivation. In August 2019, prednisone, methotrexate, and thalidomide were restarted. Ustekinumab began in June 2020 but led to perianal complications. By October 2021, the patient was hospitalized and commenced on infliximab in combination with azathioprine, but symptoms persisted and imaging showed progressive intestinal and perianal disease.

In June 2022, the patient continued to experience symptoms of abdominal pain and diarrhea. Colonoscopy revealed multiple irregular ulcers in the ileum, colon, and rectum, cobblestone‑‐like mucosal changes in the sigmoid colon, and longitudinal ulcers in the rectum. WES identified a *XIAP* truncating mutation. The patient declined HSCT. Then scRNA‑‐seq analysis showed hyperactivated JAK–STAT signaling with elevated *PDE4B* and TNF‑‐α expression in intestinal biopsies. Based on these findings, an individualized treatment regimen, combining adalimumab, upadacitinib, and dipyridamole, was initiated with the patient's informed consent. Adalimumab was used for induction and maintenance. Upadacitinib was given at 30 mg daily for 3 months, then reduced to 15 mg daily for maintenance. Dipyridamole was continued at 50 mg three times daily. The patient's symptoms gradually improved, with complete resolution of abdominal pain, diarrhea, rectal bleeding, and perianal discomfort. After 5 months of treatment, CT imaging of the abdomen indicated a notable reduction in intestinal wall thickening and inflammatory activity, and endoscopic evaluation confirmed mucosal healing of ulcers in both the small intestine and colon.

The study protocol and individualized treatment plan were approved by the Ethics Committee of the Sixth Affiliated Hospital of Sun Yat‑‐sen University (2022ZSLYEC‑‐316). The off‑‐label use of dipyridamole and upadacitinib was conducted in accordance with the hospital's pharmaceutical policies and regulatory provisions. Written informed consent was obtained from the patient for participation in the study and for the use of medications.

### 4.3 Statistical Analyses

R x64 v4.0.0 (Foundation for Statistical Computing) was applied for the statistical analysis and graphical visualization of the scRNA sequencing data. GraphPad Prism 10 (GraphPad Software) was employed to perform statistical analyses and generate graphical representations of the experimental data. Detailed descriptions of the specific statistical tests are provided in the corresponding results sections and figure legends.

## Author Contributions

ZC, YZ, Min Zhi, and WZ conceived and supervised the project. ZC, YZ, and Min Zhi wrote and revised the manuscript. Min Zhi, WW, HW, Min Zhang, XP, XY, NT, LG, HK, XZ, and WZ collected human samples and contributed to patient care. RF, LZ, JH, LH, JZ, YQ, LL, LT, QW, CN, CT, HC, and HX conducted laboratory experiments. RF, KZ, Ming Zou, and ZC performed sequencing and bio‑‐informatic analysis. JEV provided critical comments. All authors reviewed and approved the final version of the manuscript.

## Ethics Statement

The study was approved by the Medical Ethics Committee of Guangzhou Women & Children's Medical Center (No.: 2018060701) and the Medical Ethics Committee of the Sixth Affiliated Hospital of Sun Yat‑‐Sen University (No.: 2022ZSLYEC‑‐316). Biological samples were collected from all participants in accordance with ethical guidelines. The study was conducted in compliance with the International Ethical Guidelines for Biomedical Research Involving Human Subjects, as outlined in the Helsinki Declaration. Written informed consent was obtained from all participants prior to their involvement in the study.

The animal studies were approved by the Institutional Animal Care and Use Committee of Guangzhou Medical University (NO.: GY2023‑‐705) and conducted in accordance with institutional guidelines.

## Conflicts of Interest

The authors declare no conflicts of interest.

## Supporting information



Table S1 | Clinical information of subjects.
Figure S1 | Canonical marker genes for each annotated epithelial subcluster.
Figure S2 | XIAP intrinsically regulates tuft cells development
Figure S3 | *Xiap* ^−/−^ mice exhibit non‐inflammation under SPF housing conditions.
Figure S4 | Canonical marker genes for each annotated immune subcluster.
Figure S5 | XIAP‑tuft cell axis dysregulation reveals more severe CD pathology.
Figure S6 | Succinate‐ or IL‐25‐induced activation of tuft cells promote mucosal healing in *Xiap^−/−^* mice.
Figure S7 | Compositional and functional changes of myeloid and T/NK cell subsets in the XIAP‐deficient CD patient after JAK inhibitor treatment.

## Data Availability

The raw scRNA‑‐seq data from healthy controls, chronic colitis, and IBD patients were reported in our previous study [26] and have been deposited in the Genome Sequence Archive (GSA) at the Beijing Institute of Genomics, Chinese Academy of Sciences (https://ngdc.cncb.ac.cn/gsa‐human/; GSA accession number: HRA000072). The raw scRNA‑‐seq data from four XIAP‑‐deficient CD patients generated in this study have also been integrated into the same GSA dataset and are accessible under the same accession number (HRA000072). For detailed instructions on data access and application procedures, please visit the GSA portal (http://bigd.big.ac.cn/gsa‐human)
