# Supplementary material for: XIAP Deficiency Impairs Colonic Tuft Cell Development and Predisposes to Crohn's Disease
Source: MedComm (2020). 2026 Apr 25;7(5):e70745. doi: 10.1002/mco2.70745 (PMC13109868; doi:10.1002/mco2.70745)
Supplement: Supplementary file 1 — Table S1 | Clinical information of subjects. Figure S1 | Canonical marker genes for each annotated epithelial subcluster. Figure S2 | XIAP intrinsically regulates tuft cells development Figure S3 | Xiap −/− mice exhibit non‐inflammation under SPF housing conditions. Figure S4 | Canonical marker genes for each annotated immune subcluster. Figure S5 | XIAP‑tuft cell axis dysregulation reveals more severe CD pathology. Figure S6 | Succinate‐ or IL‐25‐induced activation of tuft cells promote mucosal healing in Xiap−/− mice. Figure S7 | Compositional and functional changes of myeloid and T/NK cell subsets in the XIAP‐deficient CD patient after JAK inhibitor treatment. [file MCO2-7-e70745-s001.docx]

**XIAP deficiency impairs colonic tuft cell development and predisposes to Crohn’s disease**

Rongli Fang^1, 2#^ | Wei Wang^3#^ | Li Zhang^1#^ | Jiwei Huang^2#^ | Lei Huang^1^ | Huibo Wu^3^ | Yuyin Qi^1^ | Lili Li^1^ | Liren Tan^1^ | Min Zhang^3^ | Jianheng Zhu^1^ | Xiang Peng^3^ | Kanghua Zhong^1^ | Ming Zou^1^ | Xi Yang^4^ | Qiuhua Wang^2^ | Changjun Nie^2^ | Chaorui Tang^2^ | Ning Tang^2^ | Lanlan Geng^1^ | Hanhan Chen^1^ | James E Vince^5^ | Hirokazu Kanegane^6^ | Xiaodong Zhao^4^ | Huifang Xian^1^ | Wenhao Zhou^1*^ | Min Zhi^3*^ | Yuxia Zhang^1*^ | Zhanghua Chen^1, 2*^

^1^Clinical Research Center for Pediatric Infection and Immunity, and Department of Gastroenterology, Guangzhou Women and Children’s Medical Center, Guangzhou Medical University, Guangzhou, China.

^2^Department of Comprehensive Experimental Center, Guangxi Clinical Research Center for Obstetrics and Gynecology, Liuzhou Hospital of Guangzhou Women and Children’s Medical Center, Liuzhou, China.

^3^Department of Gastroenterology, and Biomedical Innovation Center, The Sixth Affiliated Hospital, Sun Yat-Sen University, Guangzhou, China.

^4^Department of Rheumatology and Immunology, Children’s Hospital of Chongqing Medical University, Chongqing, China.

^5^The Walter and Eliza Hall Institute of Medical Research, and the Department of Medical Biology, University of Melbourne, Parkville, Vic, Australia.

^6^Department of Child Health and Development, Graduate School of Medical and Dental Sciences, Tokyo Medical and Dental University (TMDU), Tokyo, Japan.

^#^ These authors contributed equally.

The authors have declared that no conflict of interest exists.

^*^Correspondence: [zhouwenhao@fudan.edu.cn](mailto:zhouwenhao@fudan.edu.cn) (W.Z.), [zhimin@mail.sysu.edu.cn](mailto:zhimin@mail.sysu.edu.cn) (M.Z.), [yuxia.zhang@gwcmc.org](mailto:yuxia.zhang@gwcmc.org) (Y.Z.), [zhanghua.chen@gwcmc.org](mailto:zhanghua.chen@gwcmc.org) (Z.C.)

Table S1 | Clinical information of subjects


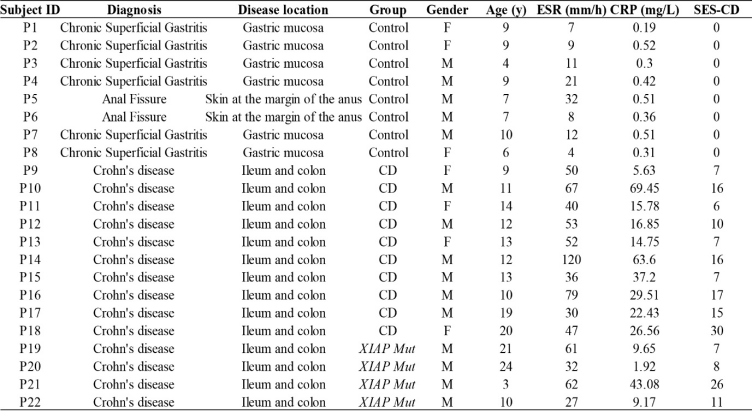


“P” represents patient, “F” represents female, “M” represents male.


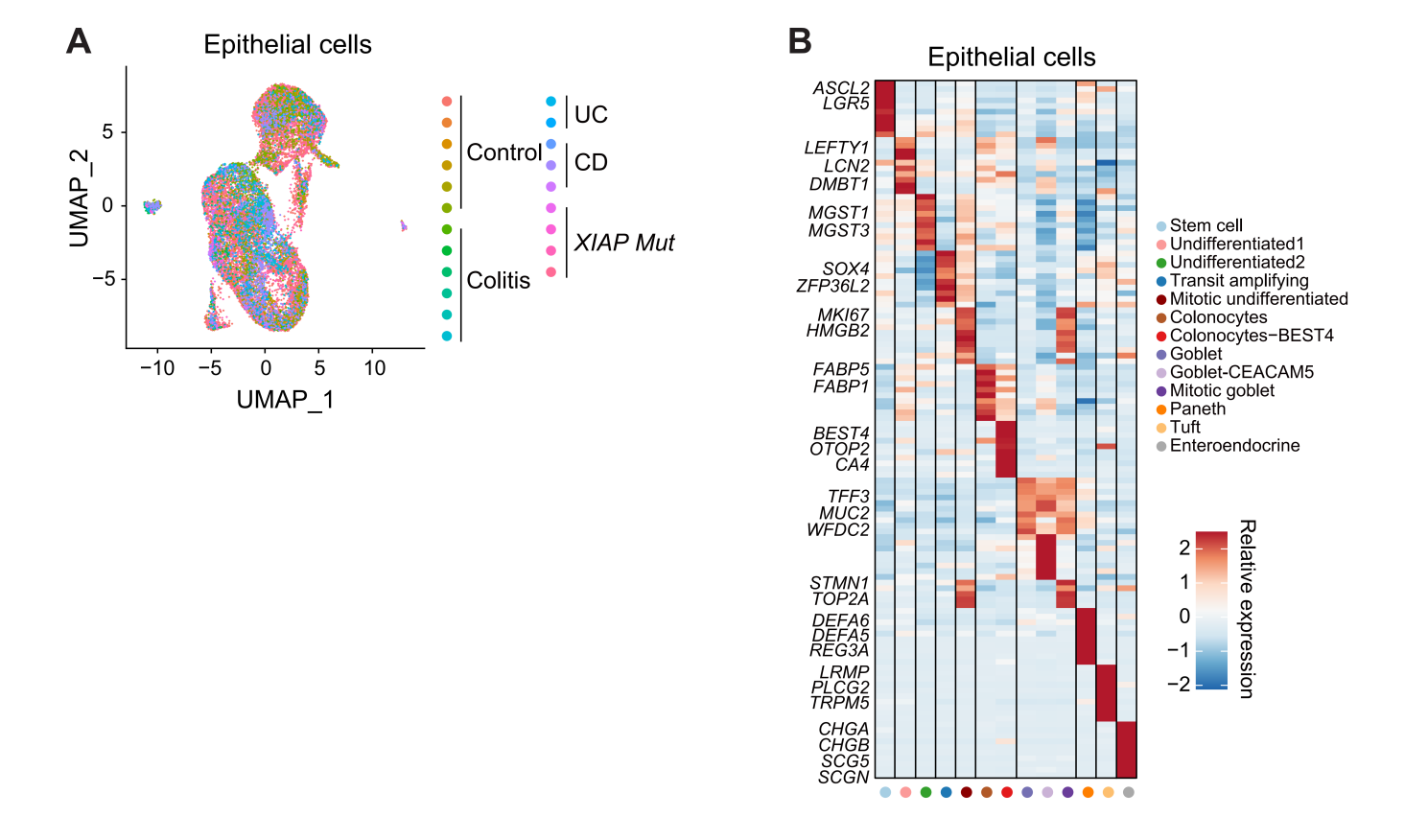


**Figure S1 | Canonical marker genes for each annotated epithelial subcluster.**

(A) UMAP plot showing the distribution of epithelial cells across individuals. Dots are colored by individual, with similar shades assigned to individuals within the same group for visual distinction. (B) Heatmap showing subtype-specific marker genes across epithelial subtypes.


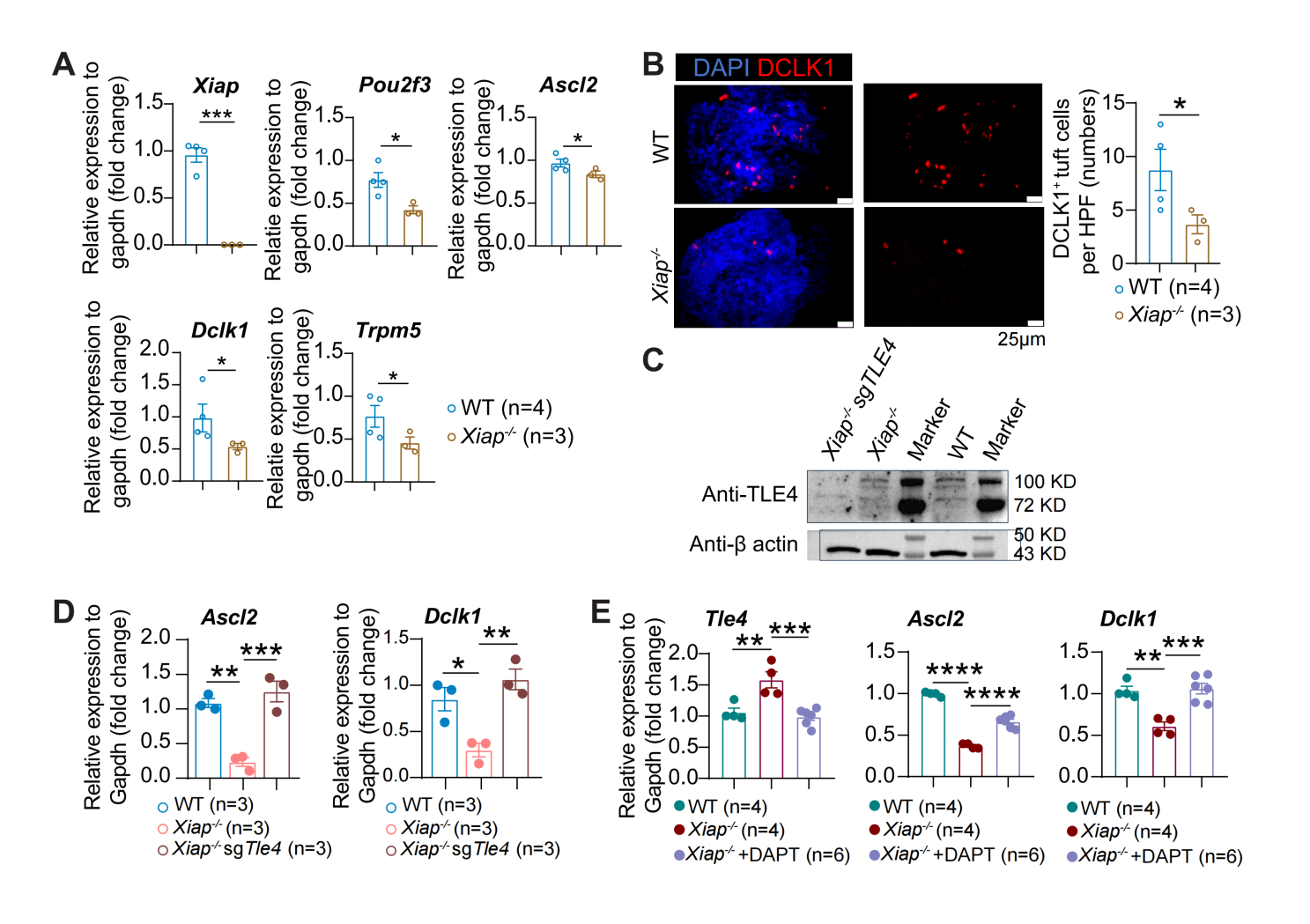


Figure S2 | XIAP intrinsically regulates tuft cells development.

(A) Bar plots comparing the expression level of *Xiap* and tuft cell marker genes in colonic organoids from WT and *Xiap^-/-^* mice. (B) Immunofluorescence staining comparing the number of DCLK1^+^ tuft cells in colonic organoids from WT and *Xiap^-/-^* mice. (C) Western blotting analysis showing reduced TLE4 protein levels under transfecting *Tle4-sgRNA* through adenovirus (MOI=100) conditions in colonic organoids derived from *Xiap^-/-^* mice. (D) Bar plots comparing mRNA expression levels of *Ascl2* and *Dclk1* in WT, *Xiap^-/-^* EV and *Xiap^-/-^ sgTle4* colonic organoids. (E) Bar plots comparing mRNA expression level of *Tle4*, *Ascl2* and *Dclk1* of WT, and *Xiap^-/-^* with or without DAPT treated colonic organoids. Data were expressed as mean ± SEM. P values were calculated using Student’s *t* test (A, B) and One-way ANOVA test (D, E). *p < 0.05, **p < 0.01, ***p < 0.001, ****p < 0.0001.


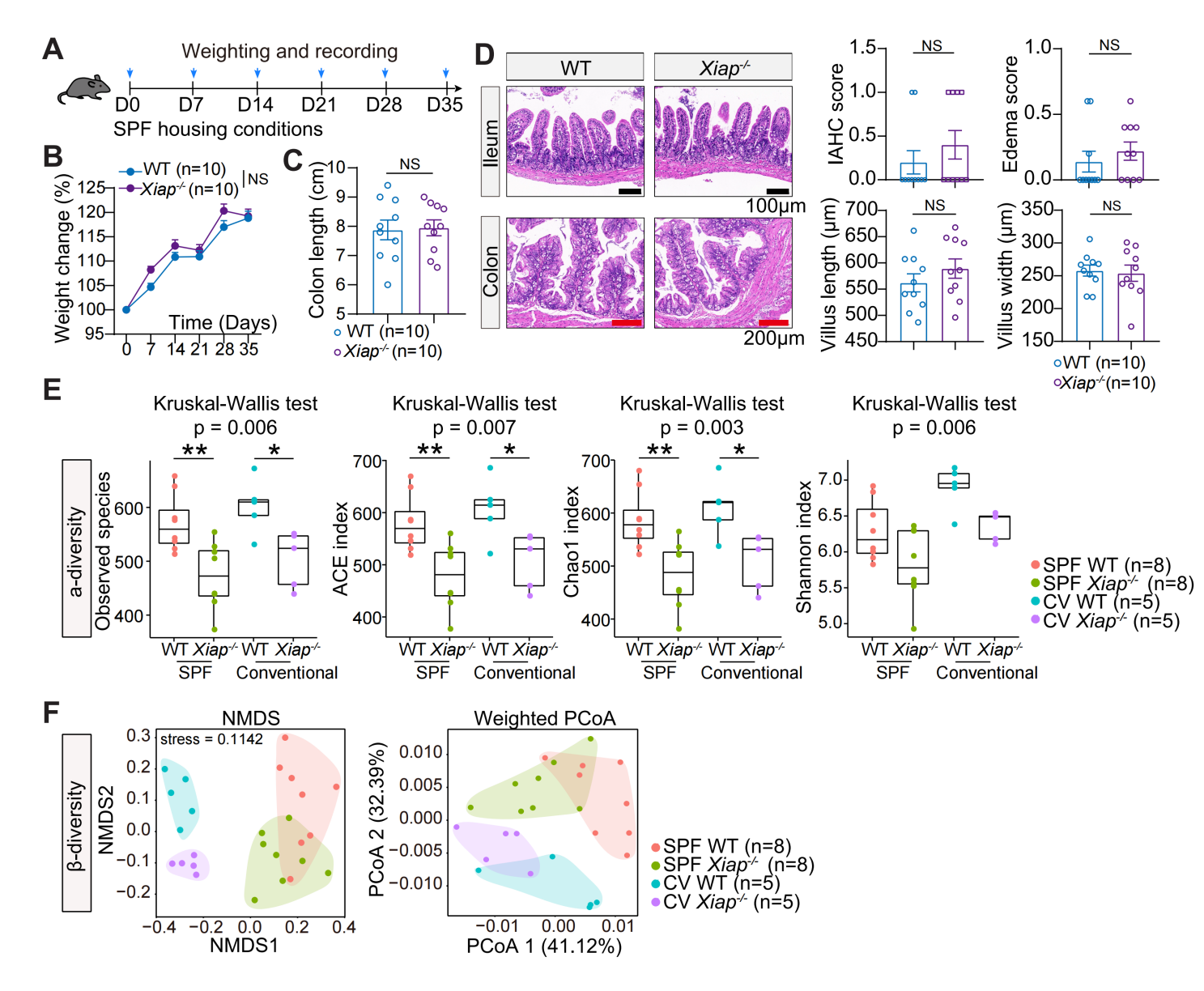


Figure S3 | *Xiap^-/-^* mice exhibit non-inflammation under SPF housing conditions.

1. Schematic illustration of SPF housing conditions for WT and *Xiap^-/-^* mice. (B) Line charts showing body weight changes in WT and *Xiap^-/-^* mice. (C) Bar plots comparing colon length between WT and *Xiap^-/-^* mice. (D) Representative H&E-stained sections from the ileum and proximal colon of WT and *Xiap^-/-^* mice. Bar plots summarizing IAHC scores, edema scores, and villus length and width. (E) Box plots comparing α-diversity metrics (observed species, ACE, Chao1 and Shannon index) between WT and *Xiap^-/-^* mice under SPF or conventional housing conditions. Boxes indicate the 25% quantile, median, and 75% quantile. (F) Non-metric multidimensional scaling (NMDS) and principal coordinates analysis (PCoA) based on weighted UniFrac distances illustrate differences in microbial community composition among WT and *Xiap^-/-^* mice under distinct housing conditions. Data were presented as mean ± SEM. Statistical analyses were performed using the Two-way ANOVA (B), Student’s *t* test (C, D) and Kruskal-Wallis’s test (E). *p < 0.05, **p < 0.01, ***p < 0.001, NS not significant.


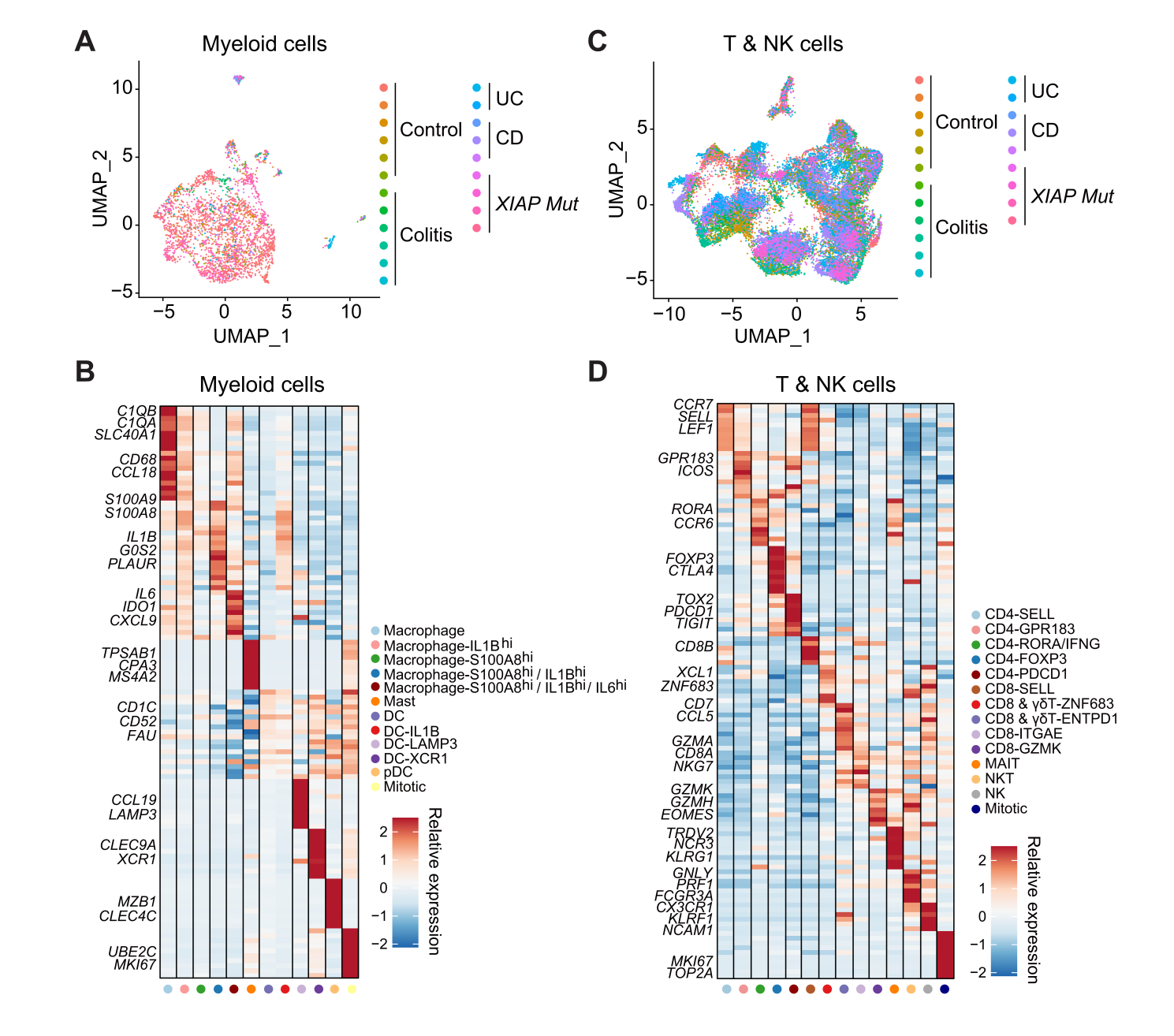


**Figure S4 | Canonical marker genes for each annotated immune subcluster.**

(A) UMAP plot showing the distribution of myeloid across individuals. Dots are colored by individual, with similar shades assigned to individuals within the same group for visual distinction. (B) Heatmap showing subtype-specific marker genes across myeloid subtypes. (C) UMAP plot showing the distribution of T/NK cells across individuals. Dots are colored by individual, with similar shades assigned to individuals within the same group for visual distinction. (D) Heatmap showing subtype-specific marker genes across T/NK subtypes.


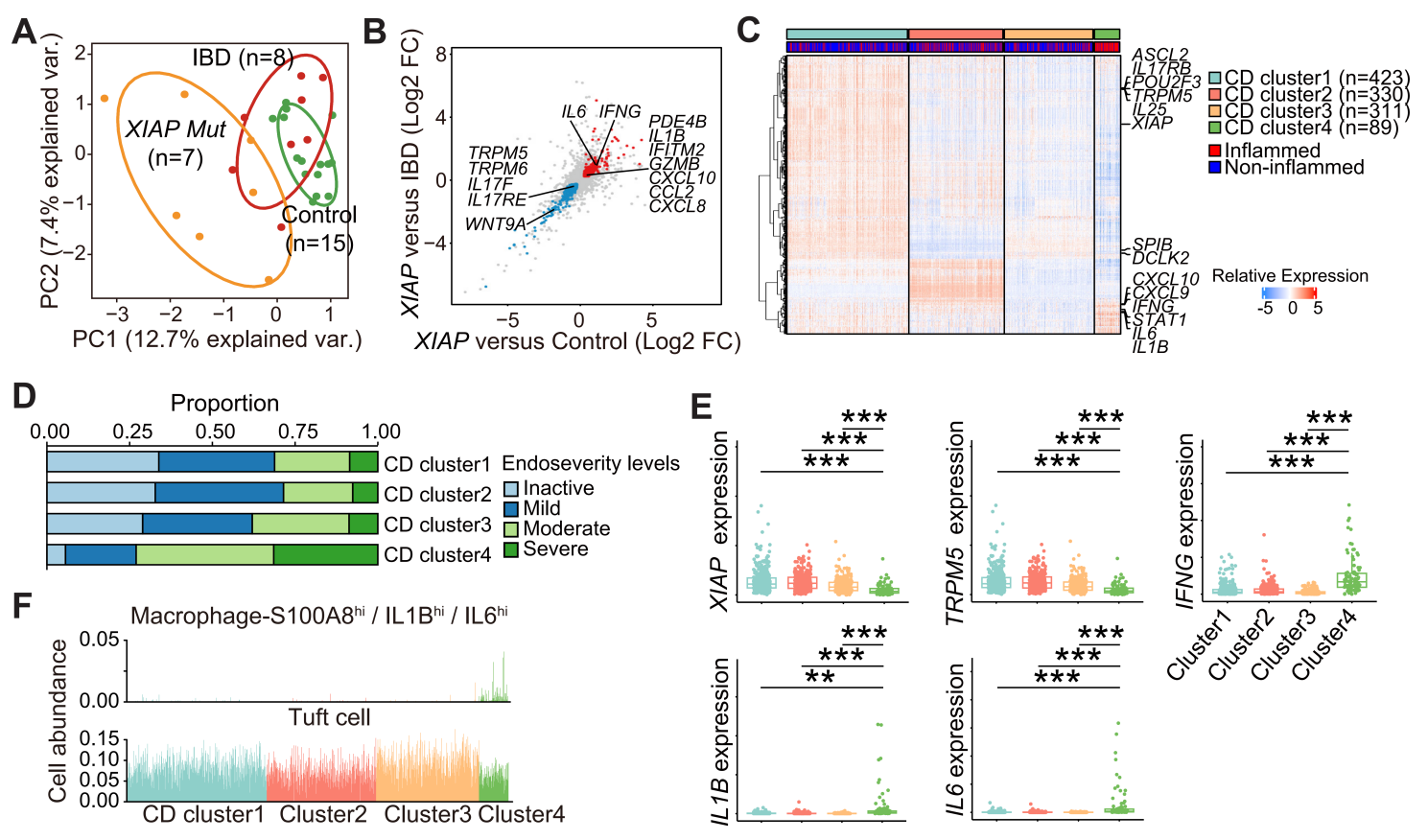


**Figure S5 | XIAP‑tuft cell axis dysregulation reveals more severe CD pathology.**

(A) Principal component (PC) analysis revealing the divergent transcriptional profiling of control (n=15), non-XIAP-deficient CD (n=8) and XIAP-deficient CD (n=7) subjects. (B) Scatter plots showing significantly up-regulated expressions of *IL6*, *IFNG*, *IL1B*, *CXCL8* and *CXCL10*, and down-regulated expressions of *TRPM5* and *TRPM6* in XIAP-deficient CD versus both control and non-XIAP-deficient CD. (C) Heatmap showing molecular stratification based on bulk RNA-seq data of 1,153 intestinal mucosae samples from 498 patients with CD. Significantly up-regulated expressions of Th1 immune response genes, and down-regulated expressions of *XIAP* and tuft cell marker genes are indicated. (D) Bar plots comparing the proportions of samples with different endoscopic severity across 4 clusters. (E) Box plots comparing expressions of *XIAP*, *TRPM5*, *IL1B*, *IL6* and *IFNG* in Cluster 1 (n=423), Cluster 2 (n=330), Cluster 3 (n=311) and Cluster 4 (n=89). Boxes indicate the 25% quantile, median, and 75% quantile. (F) Bar plots comparing cellular abundance of *S100A8/A9*^+^*IL1B*^+^*IL6^+^* inflammatory macrophages and tuft cells across different clusters. *P* values were calculated using Mann Whitney U test. **p < 0.01, ***p < 0.001.


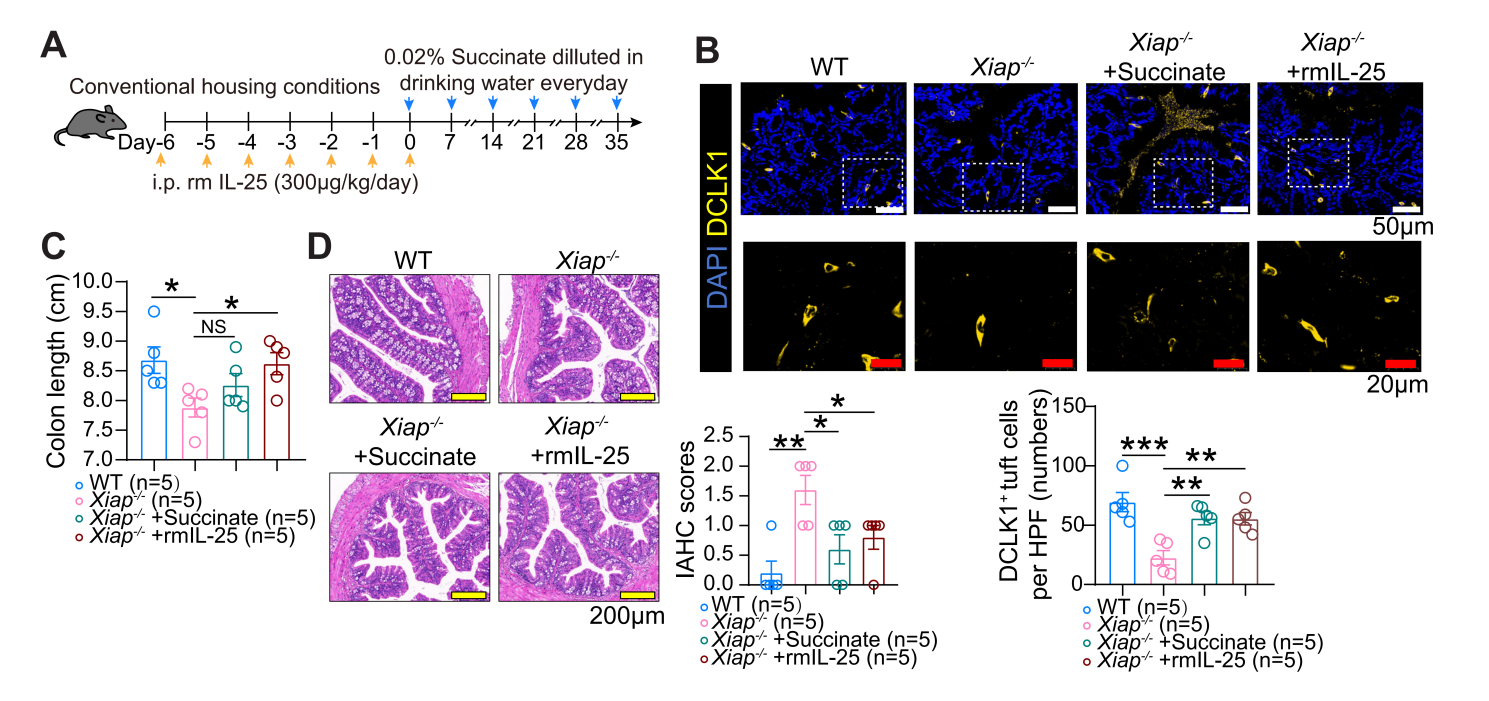


Figure S6 | Succinate- or IL-25-induced activation of tuft cells promote mucosal healing in *Xiap^−/−^* mice.

(A) Schematic diagram showing 6-week-old WT and *Xiap*^-/-^ mice were transferred from SPF to conventional housing conditions. The timeline indicates the duration in conventional housing, with arrows denoting the initiation of succinate (0.02% in drinking water) or recombinant IL-25 (300 μg/kg/d) treatment. (B) Immunofluorescence staining comparing DCLK1^+^ tuft cell abundance in the colonic mucosa of WT and *Xiap^-/-^* mice with or without succinate or IL-25 treatment. (C) Bar plots comparing colon length among WT and *Xiap^-/-^* mice with or without treatment. (D) Representative H&E-stained histological sections from proximal colons of WT and *Xiap^-/-^* mice under different treatment conditions. Bar plots summarizing IAHC scores of proximal colonic tissues across experimental groups. Data were expressed as mean ± SEM. Statistical analyses were performed using the One-way ANOVA (B-D). *p < 0.05, **p < 0.01, ***p < 0.001, NS not significant.


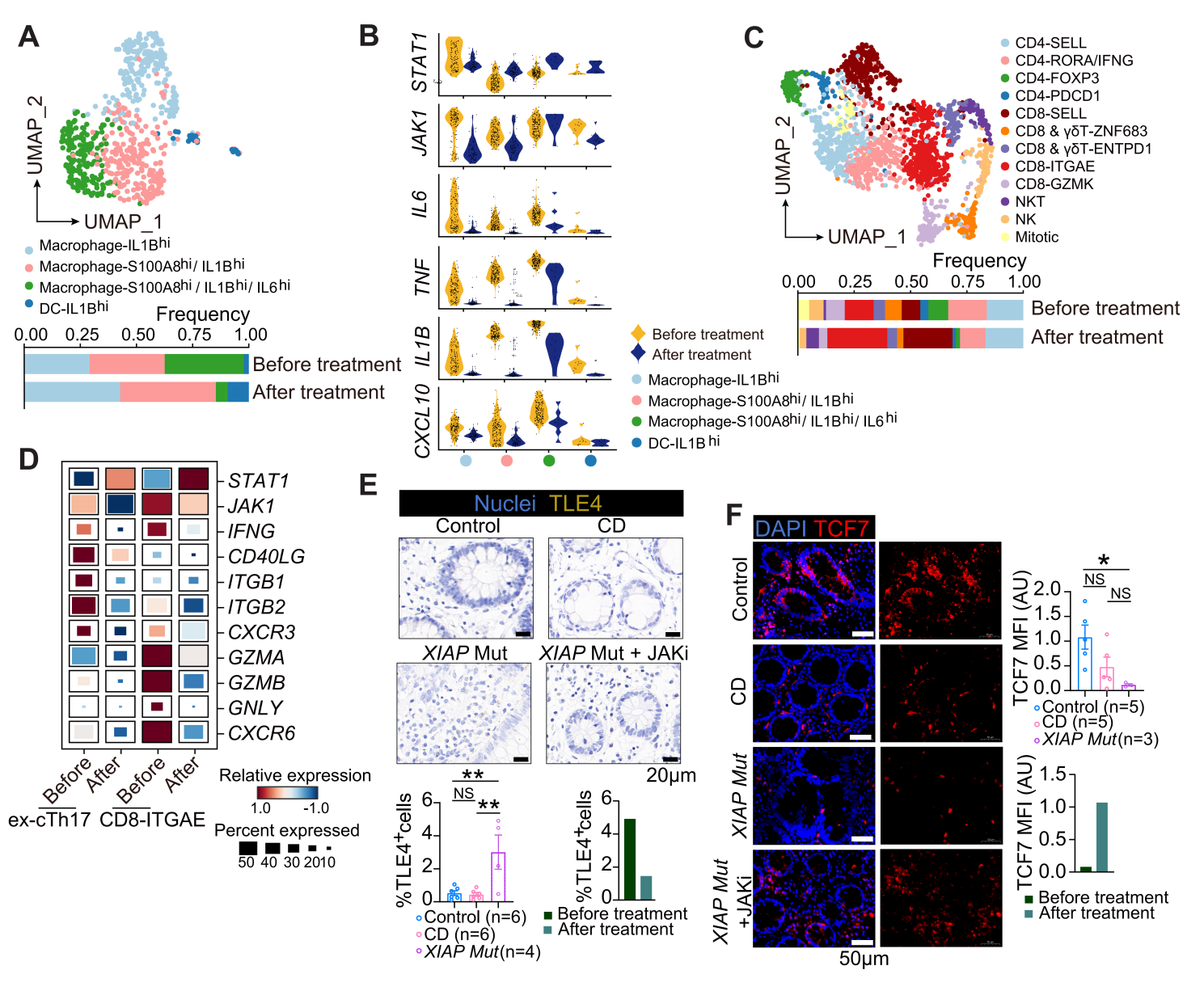


Figure S7 | Compositional and functional changes of myeloid and T/NK cell subsets in the XIAP-deficient CD patient after JAK inhibitor treatment.

(A) UMAP plot depicting 4 inflammatory macrophage and DC subsets in the XIAP-deficient CD patient before and after JAK1 inhibitor treatment. Bar plots showing the cellular proportions of these cell subsets pre- and post- treatment. (B) Violin plots comparing expression levels of Th1-related genes in the XIAP-deficient CD patient before and after treatment. (C) UMAP plot depicting 14 T and NK cell subsets in the XIAP-deficient CD patient before and after JAK1 inhibitor treatment. Bar plots showing the cellular proportions of these cell subsets pre- and post- treatment. (D) Square dot plots comparing expression of Th1 signature genes in two IFN-γ^+^ T cell subsets before and after JAK1 inhibitor treatment. Dot size reflects the proportion of cells expressing specific genes; color intensity indicates relative gene expression level. (E) IHC results comparing TLE4 expression in the colonic mucosa of control, non-XIAP-deficient and XIAP-deficient CD patients, as well as the XIAP-deficient CD patient following JAK1 inhibitor treatment. (F) Immunofluorescence staining comparing TCF7 expression in the colonic mucosa from control, non-XIAP-deficient and XIAP-deficient CD patients, and the XIAP-deficient CD patient after JAK1 inhibitor treatment. Data were expressed as mean ± SEM. Statistical analyses were performed using the One-way ANOVA (E, F). *p < 0.05, **p < 0.01, NS not significant.

Supporting materials and methods

1 | Cell culture

HEK293T cells and Caco-2 cell line were purchased from American Type Culture Collection (ATCC). Cells were cultured in DMEM containing 4.5 g/L Glucose (Gibco), supplemented with 10% FBS (Gibco), 100 units/ml penicillin (Gibco) and 100 µg/ml streptomycin (Gibco), and maintained at 37℃ in a humidified atmosphere with 5% CO_2_. For experiments purposes, cells were used between passages 2-10 and seeded at a density of 3*10^5^ cells/ml.

2 | Western blot

Total protein was extracted using a low-salt lysis buffer (50 mM HEPES, 150 mM NaCl, 1mM EDTA, 1.5 mM MgCl_2_, 10% glycerol and 1% Triton X-100) supplemented with phosphatase and protease inhibitor tablets (Roche). Proteins were mixed with 5×SDS-PAGE Sample Loading Buffer (FD), denatured by heating at 95℃ for 5 min, and then loaded onto 10% TGX Stain-Free polyacrylamide gels (Bio-Rad). Electrophoresis was performed followed by transfer of proteins to PVDF membranes (0.22 μm, Bio-Rad). Membranes were blocked with 5% skim milk in TBS-Tween 20 buffer, incubated sequentially with primary and secondary antibodies, and detected using the ChemiDoc™ Imaging System with hypersensitive chemiluminescent substrate (FD).

3 | Co-immunoprecipitation

For co-immunoprecipitation (co-IP), proteins were incubated with anti-Flag M2 Affinity gels (10 μl per sample, Sigma-Aldrich, A2220) overnight at 4℃. For endogenous co-IP, anti-TLE4 (1:200, CST, 4681S) was first conjugated to the Pierce^TM^ Protein A/G Agarose Beads (20 μl per sample, Pierce, 20421) for 4 hours at 4℃. The antibody-protein-bead complexes were washed 5 times with low-salt lysis buffer, denatured by heating, and subsequently subjected to western blot analysis.

4 | RNA extraction and quantitative real-time PCR

Total RNA was extracted from mouse colons using TRIZOL (Invitrogen) according to the manufacturer’s instructions. RNA quantity and quality were assessed using the Thermo Varioskan™ LUX multimode reader (Thermo Fisher). cDNA was synthesized by RT-PCR using HiScript II Q RT SuperMix (Vazyme, R323-01). Gene expression analysis was performed by quantitative real-time PCR using AceQ qPCR SYBR Green Master Mix (Vazyme, Q711-03) on an ABI QuantStudio 6 Real-Time PCR System (Applied Biosystems). Samples were normalized to *Gapdh* and presented as relative values.

Primers used are as follows.

*Xiap*: ACTTCCCAAGTAGTAGTCCTGTT; TATTGCCGCATGACAACTGAA.

*Lgr5*: ACCTGTGGCTAGATGACAATGC; TCCAAAGGCGTAGTCTGCTAT.

*Trpm5*: CCAGCATAAGCGACAACATCT; GAGCATACAGTAGTTGGCCTG.

*Pou2f3*: CAGCAACAAAGCACTCTACTCC; CAGTGGGCTCATCATTCCCTC.

*Ascl2*: AAGCACACCTTGACTGGTACGC; AAGTGGACGTTTGCACCTTCA.

*Mki67*: CAAGGCGAGCCTCAAGAGATA; TGTGCTGTTCTACATGCCCTG.

*Muc2*: AGGGCTCGGAACTCCAGAAA; CCAGGGAATCGGTAGACATCG.

*Chga*: CCAAGGTGATGAAGTGCGTC; GGTGTCGCAGGATAGAGAGGA.

*Lyz1*: GAGACCGAAGCACCGACTATG; CGGTTTTGACATTGTGTTCGC.

*Dclk1*: CTGGGTTAATGATGATGGTCTCC; TCCTGGTTGTTGGTAGTAGTCC.

*Tle4*: CTGGACAGGTGGTTTGGACAA; GAGGTGAAGTCATGTTGCTGC.

*Spdef*: AAGGCAGCATCAGGAGCAATG; CTGTCAATGACGGGACACTG.

*Klf4*: GTGCCCCGACTAACCGTTG; GTGCCCCGACTAACCGTTG.

*Fzd5*: GGTGTGCCAGGAAATCACG; CACAAGCGGCCAGAATTGG.

*Hes1*: CCAGCCAGTGTCAACACGA; CCAGCCAGTGTCAACACGA.

*Foxa1*: ATGAGAGCAACGACTGGAACA; TCATGGAGTTCATAGAGCCCA.

*Gapdh*: AGGTCGGTGTGAACGGATTTG; AGGTCGGTGTGAACGGATTTG.

5 | Bone marrow derived macrophage (BMDM) culture and stimulation

Following euthanasia, femurs and tibias from WT and *Xiap^-/-^* mice were aseptically removed and transferred to a biosafety cabinet. Both ends of the bones were cut and bone marrow was flushed out using a syringe and collected into 1.5mL EP tubes. Samples were centrifuged at 3,000 rpm for 10 min, and the resulting pellets were resuspended in red blood cell lysis buffer for 5 min at room temperature. The reaction was terminated by adding RPMI 1640 medium (Gibco) supplemented with 10% FBS. After a subsequent centrifugation at 1,800 rpm for 5 min, the supernatant was discarded. Cells were resuspended in RPMI 1640 medium containing 10% FBS and recombinant M-CSF (50ng/mL, PeproTech, 315-02), then seeded and cultured for 7 days to differentiate into BMDMs. To activate macrophages, cells were stimulated with the TLR4 ligand lipopolysaccharide (LPS, 100 ng/mL, Sigma, L4391). After 24 hours of stimulation, supernatants were collected for ELISA analysis.

6 | Enzyme-linked immunosorbent assay (ELISA)

IL-4, IL-5, IL-6, IL-13, IL-1β, TNF-α and Interferon-γ were quantitatively measured in mouse tissues and cell culture supernatants using commercial ELISA kits (Invitrogen). The procedures should be conducted in accordance with the manufacturer’s instructions.

7 | Histopathological staining

Paraffin-embedded tissue sections were stained with hematoxylin and eosin (H&E) staining for histopathological evaluation. The histopathological scoring criteria were as follows: Score 0, no inflammatory cell infiltration; Score 1, minimal inflammatory cell infiltration; Score 2, submucosal inflammatory cell infiltration; Score 3, transmural inflammatory cell infiltration. The number of positive cells per villus was quantified following PAS staining.

8 | Immunohistochemical and immunofluorescence staining

Following dewaxing and rehydration using xylene and graded ethanol solution, antigen retrieval was performed with pH 6.0 or pH 9.0 retrieval solution. Endogenous peroxidase was subsequently blocked with 3% hydrogen peroxide. Immunohistochemical detection was carried out using the M&R HRP/DAB Detection IHC Kit (Vazyme, HC301-02). Immunofluorescence assay was performed using the Opal 3-Plex Manual Detection Kit (Akoya Bioscience, NEL810001KT). Images were acquired using the 3D HISTECH Panoramic Scanning System (3D HISTECH). Data analysis was conducted using ImageJ to calculate the mean fluorescence intensity (MFI), and the percentage of positive cells within each defined region of interest was quantified using 3D HISTECH QuanCenter.

9 | Colonic organoid extraction

Fresh colon tissues from WT and *Xiap^-/-^* mice were repeatedly washed 15-20 times in sterile PBS. The tissues were cut into 1 mm^3^ blocks using ophthalmic scissors and transferred to 15 ml centrifuge tubes. Following three washes with sterile PBS, the supernatant was discarded and 5 ml of tissue digestion buffer was added. The samples were gently digested at 4℃ and 40 rpm for 30 minutes. After digestion, the suspension was filtered through a 100 μm cell strainer, and the filtrate was collected. Digestion was terminated by adding three volumes of culture for rinsing. The cells were then enriched by centrifugation at 300 g for 5 minutes, followed by removal of the supernatant. The crypt-containing pellets were resuspended in organoid culture medium, and three volumes of Matrigel were added and mixed thoroughly to establish a 3D organoid culture system. After culturing for 7 - 10 days in organoid growth medium, recombinant IL-13 (20 ng/ml, 72h, Thermo Fisher, 210-13) was supplemented to induce tuft cell differentiation in colonic organoids. The colonic organoid isolation kit was purchased from JFKR Organoid Bio. Co., Ltd (Shanghai, China, MNC-100-KIT).

10 | Immunofluorescence staining of colonic organoid

Colonic organoids were collected into 1.5 ml EP tubes. Following centrifugation at 300 g for 5 minutes, the supernatant was discarded and 1 ml of 4% PFA was added for fixation. All organoids were fixed at room temperature under gentle agitation (40 rpm) for 2 hours. After incubation, the organoids were allowed to settle naturally to the bottom of tube, followed by removal of the supernatant. Subsequently, 1 ml of antigen retrieval solution was added, and the samples were incubated at 95℃ for 20 minutes. After cooling to room temperature, the organoids were washed twice with IF solution buffer, and blocked at room temperature for 120 minutes. The blocking buffer was then discarded, and primary antibody-solution buffer was added for overnight at 4℃. Following this, the organoids were washed twice with IF solution buffer, and incubated with secondary antibody-solution buffer at room temperature for 60 minutes. Next, the organoids were dehydrated through a methanol series (50% and 100% methanol), air-dried naturally, and mounted using ProLong^TM^ Gold anti-fade reagent (Thermo Fisher). Immunofluorescence images were acquired using a Leica DMi8 inverted fluorescence microscope (Germany). Data analysis was performed using ImageJ to quantify mean fluorescence intensity (MFI) or cell numbers. For each individual, 3 - 5 fields of view were taken, and the average value was calculated for subsequent statistical analysis.

11 | Whole-exome sequencing analysis

For data analysis, we first performed quality control of the raw data using fastp. ^1^ Subsequently, the quality-filtered reads were aligned to the reference genome (GRCh38) using BWA software and the resulting SAM files were sorted using Picard. ^2^ Duplicate reads were marked using Picard as well. Following alignment and sorting, variant calling was performed using GATK, followed by annotation of the variant-detected VCF files using annovar. ^3,4^ The annotated variants were then filtered using SnpSift and kggseq tools to prioritize functionally relevant variants. ^5,6^ For quality assessment, bamdst was used to generate alignment statistics, and Peddy was employed to verify individual gender and relatedness. ^7^

12 | Single-cell RNA sequencing and analysis

Colonic biopsies were obtained from 4 XIAP-deficient CD patients and digested using Collagenase type IV (Gibco, 2 mg/ml), DNase I (Sigma, 10 unit/ml) and HEPES (Gibco, 0.01M) under gentle rotation. The resulting cell suspensions were processed on the 10X Genomics platform, where RNA transcripts were uniquely bar-coded within each cell and reverse-transcribed into bar-coded cDNA, followed by purification, amplification and adaptor ligation. All libraries were sequenced on the Illumina Novaseq 6000 platform.

For data analysis, the raw data were demultiplexed and aligned to the human transcriptome reference (GRCh38) using CellRanger-3.1.0 (for 10x Genomics). A raw gene expression count matrix (genes by cells) was generated for each sample and converted into Seurat object using the R package Seurat v4.0.0. ^8^ High-quality cells were defined as those expressing between 200 and 5,000 genes and containing fewer than 50,000 unique molecular identifiers (UMIs). Following quantity control, this dataset was integrated with previously generated scRNA-seq data from 6 healthy controls, 6 patients with chronic colitis, and 5 IBD patients with normal XIAP expression. The combined Seurat object was split using *SplitObject* function in Seurat, followed by identification of variable features and integration anchors across datasets via the *FindIntegrationAnchors* function*.* These anchors were then used as input for the *IntegrateData* function, which generated a Seurat object containing a batch-effects-corrected integrated expression matrix. The “integrated” assay was utilized for cell type clustering, while the original uncorrected expression matrix (“RNA” assay) was retained for the identification of differentially expressed genes (DEGs).

For cell type clustering, variable genes were selected using the Seurat’s function *FindVariableFeatures* for principal component analysis (PCA). The most significant PCs were then used for dimensionality reduction, shared-nearest neighbor graph construction, and clustering by applying the *RunUMAP*, *FindNeighbors* and *FindClusters* functions. All analyses were performed using appropriate parameters as implemented in Seurat. MAST, a hurdle model designed for zero-inflated single-cell data based on linear regression, was employed for differential expression analysis to evaluate the effects of group membership and clinical treatment. ^9^ Functional enrichment analysis of marker genes specific to each cellular subset was conducted using Metascape. ^10^

13 | Bulk RNA sequencing data analysis*.*

We re-analyzed two public datasets of bulk RNA sequencing of intestinal mucosae of control and patients with CD, to consolidate our findings about the XIAP-deficiency in CD pathogenesis. For the first dataset contained control (n = 15) subjects, non-XIAP deficient (n = 8) and XIAP-deficient (n = 7) CD patients, we identified DEGs was using the empirical Bayes moderated *t*-statistics implemented in the limma package (absolute log2FoldChange > = 0.25, *P* value < 0.05). ^11^

For the second dataset contained 1,153 intestinal mucosae samples from 498 CD patients, we used T cells, myeloid cells, and epithelial cells from our single-cell data as reference for the deconvolution analysis. Using BayesPrism, we estimated the proportions of our targeted cell subtypes in each sample of the bulk RNA dataset. ^12^ Then, we took the logarithm of the dataset and calculated the standard deviation for each gene. After sorting genes from high to low based on their standard deviation, we selected the top 1000 genes as variable features for the dataset. We then applied ConsensusClusterPlus to perform consensus clustering of the data and obtained four clusters with different transcriptional profiling. ^13^

14 | 16S rRNA sequencing and analysis

Fecal DNA from WT and *Xiap^-/-^* mice reared under SPF and conventional housing conditions were extracted. Following PCR amplification and purification, libraries were constructed and sequenced. All sequencing was performed on the Illumina Nova-seq 6000 platform.

For data analysis, the original data were subjected to quality control using Qiime2 with default parameters, including quality filtering, noise reduction, sequence concatenation and de-mosaicking. Sequences with a total abundance across all samples below 10 were removed to generate amplicon sequence variants (ASVs). Based on the resulting ASVs, multiple diversity analyses were performed, and sequencing depth was evaluated to assess library saturation. Statistical analysis of microbial community structure was conducted using taxonomic composition data.

**References:**

1. Chen S, Zhou Y, Chen Y, Gu J. fastp: an ultra-fast all-in-one FASTQ preprocessor. *Bioinformatics*. 2018;34(17):i884-i890.

2. Li H, Durbin R. Fast and accurate long-read alignment with Burrows–Wheeler transform. *Bioinformatics*. 2010;26(5):589-595.

3. McKenna A, Hanna M, Banks E, et al. The Genome Analysis Toolkit: a MapReduce framework for analyzing next-generation DNA sequencing data. *Genome research*. Sep 2010;20(9):1297-303.

4. Wang K, Li M, Hakonarson H. ANNOVAR: functional annotation of genetic variants from high-throughput sequencing data. *Nucleic acids research*. Sep 2010;38(16):e164.

5. Cingolani P, Patel VM, Coon M, et al. Using Drosophila melanogaster as a Model for Genotoxic Chemical Mutational Studies with a New Program, SnpSift. *Frontiers in Genetics*. 2012;3

6. Li M-X, Gui H-S, Kwan JSH, Bao S-Y, Sham PC. A comprehensive framework for prioritizing variants in exome sequencing studies of Mendelian diseases. *Nucleic Acids Research*. 2012;40(7):e53-e53.

7. Pedersen BS, Quinlan AR. Who’s Who? Detecting and Resolving Sample Anomalies in Human DNA Sequencing Studies with Peddy. *The American Journal of Human Genetics*. 2017;100(3):406-413.

8. Hao Y, Hao S, Andersen-Nissen E, et al. Integrated analysis of multimodal single-cell data. *Cell*. 2021;184(13):3573-3587.e29.

9. Finak G, McDavid A, Yajima M, et al. MAST: a flexible statistical framework for assessing transcriptional changes and characterizing heterogeneity in single-cell RNA sequencing data. *Genome Biology*. 2015;16(1)

10. Zhou Y, Zhou B, Pache L, et al. Metascape provides a biologist-oriented resource for the analysis of systems-level datasets. *Nature Communications*. 2019;10(1)

11. Ritchie ME, Phipson B, Wu D, et al. limma powers differential expression analyses for RNA-sequencing and microarray studies. *Nucleic Acids Research*. 2015;43(7):e47-e47.

12. Chu T, Wang Z, Pe’er D, Danko CG. Cell type and gene expression deconvolution with BayesPrism enables Bayesian integrative analysis across bulk and single-cell RNA sequencing in oncology. *Nature Cancer*. 2022;3(4):505-517.

13. Wilkerson MD, Hayes DN. ConsensusClusterPlus: a class discovery tool with confidence assessments and item tracking. *Bioinformatics*. 2010;26(12):1572-1573.
